# Psychological distress and its impact on glycemic control in patients with diabetes, Northwest Ethiopia

**DOI:** 10.3389/fmed.2025.1488023

**Published:** 2025-03-26

**Authors:** Ashenafi Kibret Sendekie, Liknaw Workie Limenh, Gizachew Kassahun Bizuneh, Asmamaw Emagn Kasahun, Samuel Agegnew Wondm, Fasil Bayafers Tamene, Ephrem Mebratu Dagnew, Kalab Yigermal Gete, Abebe Tarekegn Kassaw, Abera Dessie Dagnaw, Yabibal Berie Tadesse, Biruk Beletew Abate

**Affiliations:** ^1^Department of Clinical Pharmacy, School of Pharmacy, College of Medicine and Health Sciences, University of Gondar, Gondar, Ethiopia; ^2^Curtin Medical School, Faculty of Health Sciences, Curtin University, Bentley, WA, Australia; ^3^Department of Pharmaceutics, School of Pharmacy, College of Medicine and Health Sciences, University of Gondar, Gondar, Ethiopia; ^4^Department of Pharmacognosy, School of Pharmacy, College of Medicine and Health Sciences, University of Gondar, Gondar, Ethiopia; ^5^Department of Pharmacy, College of Health Sciences, Debre Markos University, Debre Markos, Ethiopia; ^6^School of Medicine, College of Medicine and Health Science, Bahir Dar University, Bahir Dar, Ethiopia; ^7^Department of Pharmacy, College of Medicine and Health Sciences, Woldia University, Woldia, Ethiopia; ^8^Department of Pharmaceutical Chemistry, School of Pharmacy, College of Medicine and Health Science, University of Gondar, Gondar, Ethiopia; ^9^College of Medicine and Health Sciences, Woldia University, Woldia, Ethiopia; ^10^School of Population Health, Curtin University, Bentley, WA, Australia

**Keywords:** psychological distress, glycemic control, diabetes management, diabetes distress, mental health and diabetes, emotional burden, Ethiopia

## Abstract

**Background:**

Diabetes distress is the emotional and mental burden of living with diabetes. It can include feelings of frustration, guilt, anxiety, and worry. Understanding the factors contributing to psychological distress and how it affects glycemic control can be crucial for improving patient outcomes. Therefore, this study investigated the association between psychological distress levels and glycemic control in patients with diabetes. It also identified factors associated with severity of psychological distress.

**Methods:**

A multicentre cross-sectional study was conducted among patients with diabetes at selected hospitals in Northwest Ethiopia. Psychological risk distress was measured using the Kessler 10 (K10) questionnaire, validated for this population. Glycemic control was categorized as poor and good based on patients’ current glucose records and following recommended guidelines. Logistic regression examined the association between psychological distress levels and glycemic control. Linear regression assessed the association between psychological distress score and other independent variables. *p*-value <0.05 was considered statistically significant.

**Results:**

More than half (218, 54.2%) of the participants had severe psychological distress with a 27.4 (±4.6) mean score. Patients with moderate [AOR = 1. 85, 95% CI: 1.05–3.76] and severe [AOR = 2.84, 95% CI: 1.32–7.31] distress levels significantly had poor glycemic control compared to those with no distress. BMI [*β* = 0.61, 95% CI: 0.42, 71], monthly salary [β = −0.41, 95% CI: −67, −0.25], source of healthcare cost [*β* = −0.75, 95% CI: −2.36, −0.03], SMBG practicing [*β* = −0.85, 95% CI: −1.93, −0.25], lifestyle modifications [*β* = −1.66, 95% CI: −3.21, −0.18], number of medical conditions [*β* = 0.72, 95% CI: 0.57, 2.81], number of medications [*β* = 2.26, 95% CI: 1.05, 4.57], hypoglycaemia perception [*β* = 2.91, 95% CI: 1.32, 7.01], and comorbidity and/or complications [*β* = 3.93, 95% CI: 1.08, 6.72] were significantly associated with severity of psychological distress.

**Conclusion:**

Most patients reported having moderate to severe psychological distress, which in turn, negatively impacted their glycemic control. Interventions incorporating mental health and psychosocial support should be implemented to relieve psychological distress and improve glycemic control.

## Introduction

Diabetes mellitus (DM) is a chronic metabolic disorder characterized by hyperglycemia, or high blood sugar, resulting from defects in insulin secretion, insulin action, or both (1). It is a global health crisis, affecting hundreds of millions worldwide significantly burdening healthcare systems (2, 3). Beyond its physical complications, diabetes also has a significant psychological impact, leading to distress that includes emotional, behavioral, and psychological responses to the disease (4, 5). Diabetes distress refers to the emotional and mental burden of managing diabetes, characterized by frustration, anxiety, guilt, and feelings of being overwhelmed (6).

The experience of psychological distress in diabetes is multifaceted. Patients grapple with the constant burden of managing their condition, including adhering to medication regimens, monitoring blood sugar levels, and adjusting diet and lifestyle (7, 8). This ongoing responsibility can lead to feelings of anxiety, frustration, and fear of complications (9). Additionally, the potential for social stigma associated with diabetes can exacerbate emotional distress (10). The interplay between these factors and the psychological demands of managing diabetes can create a vicious cycle, negatively impacting self-care behaviors and overall well-being.

Unlike general psychological disorders such as depression, diabetes distress is directly linked to the challenges of diabetes self-care, including medication adherence, blood sugar monitoring, and lifestyle modifications (11). Elevated distress levels are associated with poorer glycemic control, increased risk of complications, and decreased adherence to treatment plans (12–16). Furthermore, psychological distress can contribute to declining quality of life, affecting emotional well-being, social interactions, and daily functioning (12, 17). Studies worldwide also have shown a link between distress and poor glycemic control in patients with diabetes (18–23).

Identifying the factors contributing to psychological problems in patients with diabetes is critical for developing effective interventions (7). These determinants can be broadly categorized into individual, disease-related, and social aspects (24–27). Individual factors include personality traits, coping mechanisms, and social support networks, and patients with lower levels of resilience and limited social support may be more susceptible to developing distress (28, 29).

Disease-related factors encompass the type and severity of diabetes, the presence of complications, and the treatment burden. Patients with long-standing diabetes, complex treatment regimens, or existing complications may experience heightened distress (13, 26, 30). Social factors include socioeconomic status, access to healthcare, and cultural influences (31). Financial strain (32), limited access to healthcare resources (33), and social stigma (34) can increase distress. While diabetes distress stems from the daily struggles of managing the disease, diabetes stigma refers to societal judgment or discrimination against individuals with diabetes that can create distress (35).

Research on diabetes distress in Ethiopia has highlighted its prevalence and associated factors, emphasizing the role of socioeconomic and clinical factors in contributing to the experience of diabetes-related distress (26, 36, 37). In addition, existing studies have investigated factors associated with glycemic control in patients with diabetes, demonstrating relationships between sociodemographic, socioeconomic, clinical, and personal factors (38–43). However, none of these studies considered psychological parameters and their impact on patients’ glycemic control. Further research is needed to understand psychological distress levels, contributing factors, and impact on glycemic outcomes in these regions. This study investigates the association between the level of psychological distress and glycemic control among patients with diabetes. Additionally, the study demonstrated associated factors of psychological distress in this population.

## Methods

### Study design and settings

An institutional-based multicenter cross-sectional study was conducted between July and October 2022. The research was carried out at outpatient follow-up clinics of four tertiary care hospitals in Northwest Ethiopia. These hospitals were chosen at random by a lottery method. The included hospitals were the University of Gondar Comprehensive Specialized Hospital (UoGCSH), Felege Hiwot Comprehensive Specialized Hospital (FHCSH), Tibebe-Ghion Comprehensive Specialized Hospital (TGCSH), and Debre Markos Comprehensive Specialized Hospital (DMCSH). All four hospitals offer diabetic care.

### Study participants and inclusion criteria

The study included all eligible patients with diabetes, considering their willingness, capability, and ethical aspects of participation. To be included, patients met the following criteria: (I) Diagnosed with diabetes according to the American Diabetes Association (ADA) criteria using fasting blood glucose (FBG) or HbA1c and were 18 years of age or older. (II) They had been followed in the outpatient clinics of the selected hospitals for at least 3 months and received treatment, allowing them to appreciate their psychological impact more.

Patients who could not communicate questions for an interview had severe illness/complications, or had incomplete records were excluded. Additionally, pregnant mothers were not included due to ethical considerations for vulnerable populations.

### Sample size determination and sampling technique

The sample size was calculated based on the single population proportion formula with the following assumptions: *p* = 0.5 (assuming 50% of the population with psychological distress found to have poor glycemic control because of lack of previous evidence to obtain an optimum estimate for sample size), *W* = 0.05 (5% margin of error for the two-tailed type-I error), *Z* = 1.96 (at a two-sided 95% confidence level)


$$n=p{\left(1-p\right)}^{*}Z^{2}/W^{2}$$

When *n* was calculated, the sample size resulted in 384. Considering the 10% non-response rate, the final sample size was adjusted to 423. Then, to ensure representation among the selected hospitals, it was proportionally allocated based on the number of patients with diabetes identified from hospital records before the study. Consequently, we approached 155, 112, 101, and 55 samples from UoGCSH, FHCSH, DMCSH, and TGCSH, respectively.

A systematic random sampling technique was used to approach participants from selected hospitals using their unique medical identification numbers. To achieve this, an initial sample was chosen using a simple random sampling technique to ensure all patients with diabetes in the hospital records had an equal chance of being selected. Then, based on this starting point, participants were included using a sampling interval until the allocated number for each hospital was reached.

### Data collection instruments, procedures, and quality control techniques

Data were collected using a structured questionnaire administered through a face-to-face interview. Clinical data and medication information were extracted concurrently from the patient’s medical records. The questionnaire, initially developed in English, was translated into the local language (Amharic) by experts to ensure consistency.

The data collection tool consists of three parts. The first part gathers participants’ sociodemographic characteristics (age, gender, marital status, education level, employment status, average monthly household income, and social history) as well as diabetes-related clinical characteristics. This includes the duration of diagnosis, body mass index (BMI), self-monitoring of blood glucose (SMBG) status, lifestyle modification status, medications, diabetes-related complications and comorbidities, and blood glucose readings like fasting blood glucose (FBG) and HbA1c. The second section is a scale that assesses patients’ perceptions of hyperglycemia and hypoglycemia. Scores range from 0 (never) to 4 (always), with higher scores indicating a greater perceived frequency of hyperglycemia and hypoglycemia. The third section of the questionnaire is a tool used to assess patients’ psychological distress over the previous 4 weeks.

To ensure the questionnaire’s clarity, consistency, and ease of use, a pilot test was conducted with 20 patients (approximately 5% of the final sample size) at UoGCSH. These pilot participants were excluded from the main study. Experts in the field (diabetes specialists and public health researchers) who are experienced in questionnaire validation and translation also assessed the questionnaire’s face validity. Based on the pilot test and expert feedback, minor modifications were made to the questionnaire related to clarity and consistency of wording before the actual data collection began.

Four nurses and two pharmacists from the selected hospitals collected the data. Data collectors participated in a half-day training session covering the study’s purpose, the data collection instruments, and ethical considerations. After entering the medical record identification numbers of patients into Microsoft Excel 2013 to check for duplicates, they interviewed the patients and extracted relevant information from their medical records. Throughout the data collection period, completed questionnaires were reviewed daily to ensure completeness, clarity, and cleanliness to ensure logical responses, error detection, handling of missing values, and standardized data entry for accurate analysis. The supervisor strictly monitored adherence to the data collection procedures.

### Main outcome measures

This study determined the level of psychological distress and examined its association with glycemic control in patients with diabetes as the main outcome variable. The study also identified associated factors of psychological distress in patients with diabetes as a secondary outcome.

### Psychological distress

The Kessler Psychological Distress Scale (K10) assesses the frequency of mental distress in the past 4 weeks, focusing on symptoms of anxiety and depression. It uses a five-point scale where 1 indicates no symptoms and 5 indicates experiencing symptoms all the time. The K10 questionnaire has been used in the Ethiopian population and has good internal consistency, with Cronbach’s alpha of 0.83, indicating a reliable measurement of psychological distress (44). The exploratory factor analysis (EFA) revealed a two-factor structure, explaining approximately 66% of the total variance. However, confirmatory factor analysis (CFA) indicated that the best-fitting model for this setting was a unidimensional model with correlated errors. This model demonstrated an acceptable fit with a comparative fit index (CFI) of 0.90 and a root mean square error of approximation (RMSEA) of 0.10. The ten questions address common depression and anxiety symptoms like tiredness, nervousness, restlessness, hopelessness, sadness, feelings of worthlessness, and lack of motivation. Scores range from 10 (lowest) to 50 (highest), with higher scores indicating greater psychological distress. Scores below 20 suggest good mental health, 20–24 indicate mild distress, 25–29 moderate distress, and 30 or above indicate severe distress (45, 46).

### Glycemic control

Glycemic control was assessed using fasting blood glucose (FBG) levels obtained from participants’ medical records. The hospitals primarily utilized FBG for routine glycemic monitoring, and we could not find records of hemoglobin A1C (HbA1C) except for a small number of patients who had undergone testing at private clinics outside the hospital. After confirming a normal distribution, the average FBG over the past 3 months was calculated for each participant. Glycemic control was then categorized based on the American Diabetes Association (ADA) recommendations: Good glycemic control: FBG between 70 and 130 mg/dL and poor glycemic control: FBG below 70 mg/dL or above 130 mg/dL. FBG is a widely used method for assessing glycemic control in diabetes patients in research settings (40, 42).

### Data entry and statistical analysis

Before data entry, it was checked for quality, completeness, consistency, and clarity. The data was then entered into Epi Info version 8 and subsequently imported to IBM SPSS Statistics version 26 for statistical analysis.

The normal distribution of the data was examined using normal probability (P–P) plots and histograms. Continuous variables are presented as means with standard deviations (SD), while categorical variables are presented as frequencies with percentages.

Linear regression analysis was used to identify factors potentially associated with psychological distress risk score. As a continuous outcome variable, a higher score indicates an increase in distress severity. Variables with a *p*-value less than or equal to 0.2 in this initial analysis were considered for further investigation. To identify the most important factors related to psychological distress, a multivariable analysis was performed on the significant variables from the initial screening.

The quality of the final model was found statistically significant (*F* = 207.5, *p* < 0.001) and assessed using several tests: Adjusted R-squared (81.6%) indicates how well the model explains the variation in psychological distress scores, variance Inflation Factor (VIF) (< 5 for all variables) suggest that multicollinearity was not a major concern, and Durbin-Watson score was 1.4 that indicates no significant autocorrelation in the model’s residuals.

The regression results were presented using unstandardized coefficients. Beta coefficients, measured in standard deviations, represent the average change in psychological distress scores for a one-unit increase in the corresponding predictor variable. A *p*-value <0.05 (with a 95% CI) was considered statistically significant.

A separate analysis using binary logistic regression was employed to assess the association between psychological risk distress levels and glycemic control levels while adjusting for potential confounding effects of sociodemographic, socioeconomic, personal, and clinical characteristics. A *p*-value <0.05 at the 95% CI in the multivariable logistic regression analysis was considered statistically significant.

## Results

### Sociodemographic and clinical characteristics of study participants

423 patients were initially approached, and 402 (95.3% response rate) participated in this study. More than half (218, 54.2%) of the participants were male, with an average age of 55.1 years (standard deviation ±10.7). Most participants (342, 85.1%) had type 2 diabetes. At least eight out of ten participants (324, 80.6%) had comorbidities and/or complications. On average, each patient had 3.7 (±1.4) medical conditions and received 5.2(±1.6) medications (Table 1).

**Table 1:** Sociodemographic and clinical characteristics of patients with diabetes at selected hospitals in Northwest Ethiopia from July to October 2022 (*N* = 402).

Variables	Category	Frequency (%)	Mean (±SD)
Sociodemographic variables			
Sex of participants	Male	218 (54.2)	
	Female	184 (45.8)	
Residence	Urban	240 (59.7)	
	Rural	162 (40.3)	
Marital status	Single	47 (11.7)	
	Married	292 (72.6)	
	Divorced	63 (15.7)	
Educational status	Unable to read or write	53 (13.2)	
	Primary school	133 (33.1)	
	Secondary school	150 (37.3)	
	University or college and above	66 (16.4)	
Occupational status	Farmer	75 (18.7)	
	Government employee	100 (24.9)	
	Self-employed	97 (24.1)	
	Student	44 (10.9)	
	Unemployed	64 (15.9)	
	Others	22 (5.5)	
Source of healthcare cost coverage	Health insurance	291 (72.4)	
	Out of pocket	111 (27.6)	
Age of participants in years			55.1 (±10.7)
Weight of participants in Kg			65.6 (±8.3)
Monthly salary (income) (ETH. Birr)			3647.8 (±1750.5)
Clinical variables			
Smoking status	Currently smoker	68 (16.9)	
	Previously smoker	98 (24.4)	
	Non-smoker at all	236 (58.7)	
Frequent alcohol drinking habit	No	183 (45.5)	
	Yes	219 (54.5)	
Practicing SMBG	Yes	142 (35.3)	
	No	260 (64.7)	
Practicing lifestyle modification	Yes	160 (39.8)	
	No	242 (60.2)	
Physical activity status	Sedentary	182 (45.3)	
	Moderate	136 (33.8)	
	Vigorous	84 (20.9)	
Family history of diabetes	Yes	261 (64.9)	
	No	141 (35.1)	
Type of diabetes	Type 1 diabetes	60 (14.9)	
	Type 2 diabetes	342 (85.1)	
Presence of comorbidities and/or complications	Yes	324 (80.6)	
	No	78 (19.4)	
Medical conditions (comorbidities and complications)	Hypertension	289 (71.9)	
	Dyslipidemia	182 (45.5)	
	Macrovascular complications	81 (20.1)	
	Hypoglycemia in recent time	48 (11.9)	
	Microvascular complications	29 (7.2)	
	Renal disorders	21 (5.2)	
	Diabetes ketoacidosis	17 (4.2)	
	Others*	14 (3.5)	
Body mass index in Kg/M^2^			27.1 (±2.5)
Duration of diabetes since diagnosis in years			13.3 (±3.9)
Number of medical conditions			3.7 (±1.4)
Hyperglycemia perception score			2.51 (±0.5)
Hypoglycemia perception score			2.27 (±0.6)
Medications			
Medications for diabetes	Metformin plus insulin	117 (29.1)	
	Metformin plus glibenclamide	90 (22.4)	
	Metformin	76 (18.9)	
	Metformin plus glibenclamide plus insulin	61 (15.2)	
	Insulin	58 (14.4)	
Medication for comorbidities and complications	Enalapril	242 (60.2)	
	Hydrochlorothiazide	73 (18.2)	
	Amlodipine	21 (5.2)	
	Furosemide	18 (4.5)	
	Atenolol	16 (4.0)	
	Metoprolol	14 (3.5)	
	Nifedipine	14 (3.5)	
	Atorvastatin	139 (34.6)	
	Simvastatin	48 (11.9)	
	Aspirin	70 (17.4)	
	Others**	14 (3.5)	
Number of medications			5.2 (±1.6)

BMI, body mass index; SMBG, self-monitoring of blood glucose; Others*, Bacterial infections, retroviral infections, thyrotoxicosis, bronchial asthma, malaria, skin disorders; others**, antiasthmatic agents, antiretrovirals, antithyroid agents, gastrointestinal agents, antibiotics.

### Psychological distress outcomes

More than half (218, 54.2%) of the participants reported severe psychological distress. The average K10 score was 27.4 (±4.6) out of 50 (Table 2).

**Table 2:** Levels of psychological distress among patients with diabetes at selected hospitals in Northwest Ethiopia from July to October 2022 (*N* = 402).

	Category	Frequency (%)	K10 scale mean (±SD) score
Overall level of psychological distress	Well mental health	40 (10)	**27.4 (±4.6)**
	Mild problems	67 (16.7)	
	Moderate problems	77 (19.2)	
	Sever problems	218 (54.2)	

Bold indicate, the overall K10 mean score.

### Factors of psychological distress in patients with diabetes

The multivariate regression analysis showed that the BMI of patients, monthly salary, sources of healthcare cost coverage, SMBG practice status, lifestyle modification status, number of medical conditions, number of medications, hypoglycemic perception, and comorbidities and/or complications were associated with psychological distress level.

Patients with higher body mass index [*β* = 0.61, 95% CI: 0.41, 0.71], higher number of medical conditions [*β* = 0.72, 95% CI: 0.57, 2.81], higher number of medications [*β* = 2.26, 95% CI: 1.05, 4.57], higher hypoglycemia perception score [*β* = 2.91, 95% CI: 1.32, 7.01], and those patients with comorbidity and/or complications [*β* = 3.93, 95% CI: 1.08, 6.72] had severe psychological distress score compared with their counterparts. On the other hand, patients with higher monthly salaries [*β* = −0.41, 95% CI: −67, −0.25], those who used health insurance-based healthcare cost coverage [*β* = −0.75, 95% CI: −2.36, −0.03], and those patients practiced SMBG [*β* = −0.85, 95% CI: −1.93, −0.25] and lifestyle modifications [*β* = −1.66, 95% CI: −3.21, −0.18] were significantly associated lower likelihood of psychological distress score (Table 3).

**Table 3:** Factors associated with psychological risk distress score among patients with diabetes at selected hospitals in Northwest Ethiopia from July to October 2022 (*N* = 402).

Variables	Categories	β-coefficient (95% CI)		*p*-value
		Simple linear regression	Multivariate regression	
Age	–	0.023 (−0.04, 0.73)	0.019 (−0.002, 0.621)	0.455
Weight	–	0.011 (0.008, 0.017)	0.013 (−0.012, 0.006)	0.312
BMI (kg/m^2^)	–	0.747 (0.354, 1.721)	0.614 (0.416, 0.714)	< 0.01*
Number of medical conditions	–	0.876 (0.234, 3.304)	0.718 (0.573, 2.809)	< 0.001*
Number of medications	–	1.712 (0.372, 4.056)	2.264 (1.051, 4.573)	< 0.001*
Hypoglycemia perception score	–	2.571 (1.238, 7.470)	2.913 (1.322, 7.006)	< 0.001*
Monthly salary	–	−0.712 (−1.003, −0.243)	0.413 (−0.671, −0.254)	0.021*
Source of healthcare cost coverage	Health insurance Out of pocket	−0.231 (−0.450, −0.154) Reference	−0.75 (−2.364, −0.033) Reference	0.001*
Practicing SMBG	Yes No	−2.412 (−4.201, −0.234) Reference	−0.852 (−1.934, −0.251) Reference	0.013*
Practicing lifestyle modification	Yes No	−1.724 (−3.756, −0.371) Reference	−1.657 (−0.324, −0.183) Reference	0.01*
Presence of comorbidity and/or complications	Yes No	4.132 (1.7314, 8.210) Reference	3.932 (1.076, 6.724) Reference	< 0.001*

CI, confidence interval; BMI, body mass index; SMBG, Self-monitoring of blood glucose; * indicated *p*-value < 0.05.

### Level of glycemic control

Most of the patients, 306 (76.1%) had poor glycemic control and the average level of blood glucose measured in FBG was 176.5(±51.6) mg/dl (Table 4).

**Table 4:** Level of glycemic control among patients with diabetes at selected hospitals in Northwest Ethiopia from July to October 2022 (*N* = 402).

Variables	Category	Frequency (%)	Mean (±SD)
Level of glycemic control	Good	96 (23.9)	
	Poor	306 (76.1)	
Fasting blood glucose (mg/dl) levels	–	–	176.5 (±51.6)

### Association between psychological distress and glycaemiac control

After adjusting other variables, the multivariable logistic regression analysis showed that the level of psychological distress had a significant association with glycemic control. Consequently, patients with moderate [AOR = 1. 85, 95% CI: 1.05–3.76] and severe [AOR = 2.84 (1.32–7.31)] psychological distress problems had significantly poor glycemic control compared with those who were mentally well patients (Table 5).

**Table 5:** Association between level of psychological distress and glycemic control among patients with diabetes at selected hospitals in Northwest Ethiopia from July to October 2022 (*N* = 402).

Variables	Category	Level of glycemic control		AOR (95% CI)	*p*-value
		Good	Poor		
Level of psychological problems	Well mental health	17	23	1	
	Mild problems	15	52	1. 083 (0.764–5.431)	0.131
	Moderate problems	22	55	1. 850 (1.045–3.762)	0.03*
	Sever problems	42	176	2.835 (1.321–7.307)	0.01*

AOR = adjusted odds ratio, CI = confidence interval, * Statistically significant.

## Discussion

This study investigated the level of psychological distress and its impact on glycemic control in patients with diabetes. The findings revealed a high prevalence of severe psychological distress among participants with diabetes. The severity of psychological distress was significantly associated with various factors including BMI, socioeconomic indicators (monthly salary, healthcare coverage), diabetes self-management practices (SMBG, lifestyle modifications), number of medical conditions and medications, hypoglycemia perception, and presence of comorbidities/complications. Patients with moderate or severe psychological distress were more likely to have poor glycemic control compared to those with good mental health.

The current study’s prevalence of high severe psychological distress in patients with diabetes aligns with existing research (47). These results underscore the significant mental health burden faced by individuals with diabetes, which may be attributed to the chronic nature of the disease, the demands of self-management, and the fear of complications (7, 8). Additionally, social stigma associated with diabetes could contribute to heightened distress (10). However, another study reported a relatively lower prevalence of severe diabetes distress (48). This difference may be attributed to variations in the sociodemographic and clinical backgrounds of the study populations. Additionally, differences in the availability and utilization of psychosocial healthcare services across healthcare facilities could be another contributing factor. The high prevalence of severe psychological distress in the current study may not only affect patients’ quality of life but can also impact treatment adherence and overall health outcomes. Given the public health significance of this issue, early identification and routine screening for psychological distress should be integrated into diabetes care. Furthermore, incorporating mental health services and exploring the benefits of psychosocial interventions are essential steps toward improving both psychological well-being and glycemic control in patients with diabetes.

Consistent with previous studies (49, 50), this study identified a significant association between high BMI and psychological distress in patients with diabetes. This could be because of some mechanisms. Body image concerns are among the mechanisms (51). People with high BMI might experience social stigma and dissatisfaction with their bodies, leading to distress. The other reason is because of underlying health issues. High BMI can be a marker for underlying health conditions that contribute to both physical and mental health problems. Inflammation which is related to chronic low-grade inflammation associated with obesity might hurt mental well-being (52). The relationship between BMI and psychological distress might be bidirectional (53). Psychological distress can also lead to unhealthy behaviors like overeating, contributing to weight gain. Future research may be needed to explore the underlying mechanisms of this association. The findings may suggest the importance of addressing both physical and mental health aspects of diabetes. Thus, considering potential strategies for weight management in patients with diabetes that consider the mental health aspects, such as incorporating cognitive-behavioral therapy alongside dietary and exercise interventions will be important.

The association between socioeconomic factors monthly salary, healthcare coverage and psychological distress in patients with diabetes aligns with a growing body of research that highlights social determinants of health (31, 54). This could be a financial strain. Those people with lower incomes might face financial difficulties affording mental healthcare, medication, and healthy food choices. Particularly those patients with diabetes and comorbidities could have multiple health complications that need a higher number of medications. This in turn leads patients to become distressed more and more. Lack of access to healthcare could also play a role that limited access to mental health specialists, which can create barriers to receiving proper treatment for distress. In addition, lower socioeconomic status can be associated with greater exposure to social stressors like discrimination, unemployment, and poor living conditions, which can contribute to mental health problems. The findings suggest potential barriers to mental healthcare access and resources. This needs to be addressed to improve overall well-being through expanding access to affordable mental healthcare through insurance coverage or public programs, and community-based mental health programs that cater to underserved populations. Addressing social determinants of health through policies promoting economic opportunity and social justice is needed. In addition, emphasizes the importance of considering the social context of a patient’s life when managing diabetes needs to be considered. Advocate for healthcare providers to screen for social determinants of health and connect patients with relevant resources, such as financial assistance programs or social support services.

This study found that patients who practiced self-management were found to have a lower likelihood of experiencing psychological distress. The finding is in line with previous studies (55). The link between self-management practices (SMBG, lifestyle modifications) and distress highlights the importance of holistic diabetes management programs that address both physical and mental health. Studies also show a positive correlation between regular blood sugar monitoring and better glycemic control (40, 42). This empowers patients and reduces feelings of helplessness. Similarly, stress management is among the crucial aspects of diabetes management like healthy eating habits and regular physical activity (56). These require ongoing effort but can significantly improve overall well-being. Therefore, this study may suggest applying holistic self-management and lifestyle programs that integrate diabetes education with mental health support and stress management techniques. This empowers patients to manage their condition effectively and improve their overall quality of life by incorporating a holistic approach to diabetes self-management including psychological support to improve self-efficacy, reduce distress, and better glycemic control than traditional programs (57, 58).

Comorbidities and complications, number of medical conditions and medications, and hypoglycemic perceptions: The association between comorbidities/complications and distress is likely due to the additional burden of managing multiple health conditions. People with diabetes are more likely to have co-existing conditions like hypertension, heart disease, and kidney disease (39). In this study, most of the patients had comorbidities. These comorbidities further complicate management and increase the treatment burden, in turn, this causes distress for patients with diabetes. Chronic hyperglycemia can lead to various complications affecting the eyes, nerves, kidneys, and feet. The fear of these complications and the ongoing management needs can be a significant source of stress (9). In addition, managing multiple chronic conditions often requires numerous medications (polypharmacy), leading to complex regimens and potential medication adherence issues (40). This experience causes distress for patients. The fear of low hypoglycemia is a major concern for patients with diabetes. This fear can lead to anxiety and avoidance of necessary medications, impacting overall glycemic control (59). While traditional diabetes management often prioritizes blood sugar control, studies emphasize the importance of addressing emotional aspects (29, 60). Programs integrating psychological support, medication management, and comorbidity education can significantly enhance patient well-being and reduce distress (61, 62).

The study disclosed that patients who had moderate to severe psychological distress were found to have poor glycemic control. Studies also have shown a clear link between psychological distress, such as anxiety and depression, and poorer glycemic control in patients with diabetes (18–22, 63–65). This can manifest as difficulties with medication adherence, healthy eating habits, and overall self-management. Distress can impact self-care behaviors through various mechanisms. It can lead to decreased motivation, increased stress hormones affecting blood sugar levels, and difficulty managing emotions that might lead to unhealthy coping mechanisms like overeating (29). This negative impact of psychological distress linked to poor glycemic control underscores the importance of addressing mental health as part of diabetes management to improve overall health outcomes. Research suggests that diabetes-specific distress, encompassing feelings of burden, fear, and frustration related to managing the disease, is a significant contributor to poor glycemic control (14, 63). This might be bidirectional in that poor glycemic control itself can also contribute to psychological distress (66). Fluctuations in blood sugar levels can lead to fatigue, mood swings, and difficulty concentrating, which can exacerbate anxiety and depression. Studies have shown the effectiveness of interventions that address both psychological distress and diabetes management skills (22, 67, 68). These interventions can lead to improved glycemic control and reduced emotional burden. Generally, psychological distress and glycemic control are intricately linked to diabetes. Distress can hinder self-management efforts, leading to poorer glycemic control, while poor glycaemia control can further exacerbate distress. Healthcare professionals need to recognize this cycle and implement strategies to address both physical and mental health aspects of diabetes.

In general, diabetes-related psychological distress refers to the emotional and psychological burden experienced by individuals managing diabetes, often stemming from the complexities of self-care, disease progression, and associated health challenges. This study also highlights that most patients experience moderate to severe psychological distress which significantly impacts glycemic control. Key contributing factors include sociodemographics, socioeconomic status, self-management practices, and clinical characteristics. Given these findings, effective diabetes management should integrate mental health and psychosocial support to address these underlying stressors and improve overall health outcomes.

### Strengths and limitations of the study

Despite the study’s valuable contribution to both practitioners and patients by aiding treatment decisions through consideration of mental health interventions, it has some limitations. Psychological burden was assessed using self-reported measures, which, while reliant on patient honesty, may lead to potential overestimation or underestimation of outcomes. Additionally, the study design does not allow for a definitive conclusion about the cause-and-effect relationship between glycemic levels and psychological distress risk levels.

The short-term nature of the study limits generalizability, as changes in mental health status may vary over a longer period. Furthermore, the generalizability is constrained by sample characteristics, as the majority of participants had type 2 diabetes. The study settings used FBG instead of HbA1C for routine glycemic monitoring; however, FBG may not accurately reflect long-term glycemic control as HbA1C does. Therefore, future research using prospective follow-up on a large population will be recommended to explore the association of different variables with psychological distress and the impact of psychological distress on glycemic control in patients with diabetes.

Future research with a prospective follow-up on a larger population is recommended to explore the associations between various factors and psychological distress, as well as the impact of psychological distress on glycemic control in patients with diabetes.

### Implications and contributions to the field

The study underscores the critical role of mental health in managing diabetes. It highlights that psychological distress is a significant factor affecting glycemic control, suggesting that traditional approaches focusing solely on blood sugar management may be insufficient. The findings emphasize integrating mental health and psychosocial support into diabetes care. This approach can help alleviate the psychological burden on patients, leading to improved glycemic control and overall well-being. The study also has implications for healthcare policy and guidelines. It suggests that policymakers and healthcare providers should consider incorporating mental health and psychosocial interventions into diabetes care programs to address the needs of patients experiencing psychological distress.

The study contributes to a growing body of research that recognizes the complex interplay between physical and mental health in diabetes. It expands our understanding of the factors influencing glycemic control, beyond just blood sugar levels. The findings provide evidence for the need for mental health and psychosocial interventions in improving glycemic control and overall patient outcomes in patients with diabetes. This can inform the development and implementation of evidence-based interventions to address the needs of this population. The study conducted in Northwest Ethiopia can contribute to understanding health disparities and the specific needs of patients in different regions. It highlights the importance of culturally sensitive and context-appropriate interventions to address mental health and diabetes in diverse populations.

## Conclusion

This study found that most patients with diabetes reported to have moderate to severe psychological distress, which significantly impacted glycemic control. The severity of psychological distress was significantly associated with factors such as sociodemographics, socioeconomic status, self-management practices, and clinical characteristics like co-existing conditions, number of medications, and hypoglycemia perception. Therefore, diabetes management and interventions should address these contributing factors and should be integrated with mental health and psychosocial support.

## Data Availability

The original contributions presented in the study are included in the article/supplementary material, further inquiries can be directed to the corresponding author.
